# Pretreatment ^18^F‐FDG uptake heterogeneity may predict treatment outcome of combined Trastuzumab and Pertuzumab therapy in patients with metastatic HER2 positive breast cancer

**DOI:** 10.1186/s40644-023-00608-0

**Published:** 2023-09-19

**Authors:** Guang Ma, Shuhui You, Yizhao Xie, Bingxin Gu, Cheng Liu, Xichun Hu, Shaoli Song, Biyun wang, Zhongyi Yang

**Affiliations:** 1 Department of Nuclear Medicine, Fudan University Shanghai Cancer Center, Shanghai, 200032 China; 2 Department of Oncology, Shanghai Medical College, Fudan University, Shanghai, 200032 China; 3 Center for Biomedical Imaging, Fudan University, Shanghai, 200032 China; 4 Shanghai Engineering Research Center of Molecular Imaging Probes, Shanghai, 200032 China; 5 Department of Breast Cancer and Urological Medical Oncology, Fudan University Shanghai Cancer Center, Shanghai, 200032 China; 6 Department of Medical Oncology, Zhongshan Hospital Fudan University, Shanghai, China

**Keywords:** Heterogeneity of ^18^F-FDG uptake, Prognosis, Metastatic HER2 positive breast cancer, Dual target therapy

## Abstract

**Objective:**

Intra-tumoral heterogeneity of ^18^F‐fluorodeoxyglucose (^18^F‐FDG) uptake has been proven to be a surrogate marker for predicting treatment outcome in various tumors. However, the value of intra-tumoral heterogeneity in metastatic Human epidermal growth factor receptor 2(HER2) positive breast cancer (MHBC) remains unknown. The aim of this study was to evaluate ^18^F‐FDG uptake heterogeneity to predict the treatment outcome of the dual target therapy with Trastuzumab and Pertuzumab(TP) in MHBC.

**Methods:**

Thirty-two patients with MHBC who underwent ^18^F-FDG positron emission tomography/computed tomography (PET/CT) scan before TP were enrolled retrospectively. The region of interesting (ROI) of the lesions were drawn, and maximum standard uptake value (SUVmax), mean standard uptake value (SUVmean), total lesion glycolysis (TLG), metabolic tumor volume (MTV) and heterogeneity index (HI) were recorded. Correlation between PET/CT parameters and the treatment outcome was analyzed by Spearman Rank Test. The ability to predict prognosis were determined by time‐dependent survival receiver operating characteristic (ROC) analysis. And the survival analyses were then estimated by Kaplan‐Meier method and compared by log‐rank test.

**Results:**

The survival analysis showed that HI_50%_ calculated by delineating the lesion with 50%SUVmax as threshold was a significant predictor of patients with MHBC treated by the treatment with TP. Patients with HI_50%_ (≥ 1.571) had a significantly worse prognosis of progression free survival (PFS) (6.87 vs. Not Reach, *p* = 0.001). The area under curve (AUC), the sensitivity and the specificity were 0.88, 100% and 63.6% for PFS, respectively.

**Conclusion:**

^18^F-FDG uptake heterogeneity may be useful for predicting the prognosis of MHBC patients treated by TP.

**Supplementary Information:**

The online version contains supplementary material available at 10.1186/s40644-023-00608-0.

## Introduction

Breast cancer(BC) is the most prevalent malignancy, accounting for 31% of female cancer cases, and the second-leading cause of cancer death in women [1]. Human epidermal growth factor receptor 2(HER2) is a receptor tyrosine-protein kinase, which is highly expressed in about 15% of patients with BC [2, 3]. This subtype of BC is considered aggressive and is associated with a poor prognosis [4]. However, monoclonal antibodies against HER2 protein can significantly improve the prognosis of patients with HER2 positive BC [5]. Research shows that Trastuzumab mainly binds to subdomain IV of HER2 extracellular domain, while Pertuzumab mainly binds to subdomain II of HER2 extracellular domain to exert their antitumor effect [6, 7]. The combination therapy with Trastuzumab and Pertuzumab(TP) in the treatment of MHBC patients can combine different HER2 epitopes to more comprehensively target HER2 receptor, which produces greater antitumor activity than either agent alone [8]. A recent double-blind, randomized, placebo-controlled phase 3 clinical trials showed that an 8-year overall survival rate was 37% for patients with MHBC treated by the dual-target therapy with TP [9].

At present, the dual anti-HER2 therapy with TP and docetaxel/paclitaxel is the first line of treatment for HER2 positive metastatic or recurrent breast cancer according to the National Comprehensive Cancer Network (NCCN) Guidelines Version 4.2023 [10]. However, there are still some patients with MHBC who cannot benefit from TP. There is an urgent need for biomarker to early select patients with HMBC who can benefit from anti-HER2 therapy before treatment for oncologists.

As a functional imaging technique, PET/CT has been widely used in the field of clinical oncology, with indications including tumor diagnosis, treatment response evaluation, recurrence monitoring, etc. [11–13]. Some studies have shown that pretreatment ^18^F-FDG PET/CT has a good predictive value in evaluating the efficacy of treatment for tumors, and the predictive value of intra-tumoral heterogeneity is particularly prominent [14]. PET/CT parameter heterogeneity index(HI) was first proposed by Johannes Salamon, which was applied to evaluate the characterization of peripheral nerve sheath tumors in neurofibromatosis [15]. Research shows HI has been shown to have good prognostic efficacy in various tumors in many studies [16–19]. We have previously showed that the HI of ^18^F-PET/CT uptake can potentially predict the treatment outcome, including breast cancer [16, 20], nasopharyngeal carcinoma [14, 17, 18]. However, the HI of ^18^F-FDG PET/CT imaging has not been reported to predict the prognosis of patients with MHBC treated by the TP, and its predictive value is unclear.

Hence, the aim of this study is to evaluate the predictive ability of heterogeneity in ^18^F-FDG uptake to predict the therapeutic outcomes of dual target therapy with TP for MHBC.

## Methods

### Patient cohort and treatment

From June 2019 to July 2022, patients with MHBC who received ^18^F-FDG PET/CT scan 1 month prior to dual anti-HER2 therapy with TP plus chemotherapy in Fudan University Shanghai Cancer Center were retrospectively enrolled. Exclusion criteria included: (1) Individuals with incomplete medical histories. (2) Patients who have other primary tumors. Most patients received docetaxel [75 mg/m2, d1, every 21-day cycle] or paclitaxel [80 mg/m2, d1, every 7-day cycle] plus an 8-mg/kg loading dose of Trastuzumab, followed by a 6-mg/kg maintenance dose, and 840-mg Pertuzumab loading dose followed by a 420-mg maintenance dose every 3 weeks as systemic therapy regimen for advanced disease. Patients received CT or MRI every 1–2 months. PET/CT and SPECT/CT are also performed as needed. Tumor response was assessed by the physician according to the Response Evaluation Criteria in Solid Tumors 1.1 (RECIST1.1) during treatment until disease progression.

All patients who met this criterion were included in subsequent analysis. Imaging data, complete medical history, tumor evaluation, and follow‐ups were retrieved from a medical electronic database system. The treatment effect was assessed using 1-year progression‐free survival (PFS). This study was approved by the institutional review board of Shanghai Cancer Center. Considering its retrospective nature, a waiver of informed consent was granted.

### ^18^F‑FDG PET/CT imaging

The radiochemical purity of ^18^F-FDG was over 95%, which was generated automatically by the cyclotron (CTIRDS Eclipse ST, Siemens, Knoxville, TN). All patients fasted for at least 6 h and their blood glucose was under 140 mg/dL before intravenous injection of FDG at a dose of 3.7 MBq/kg, followed by lying quietly for 1 h. And the ^18^F-FDG PET/CT scans ranged groin to skull base. The PET/CT acquisition parameters were as follows: First, CT scanning was performed with 120 kV, 80–250 mA, pitch 3.6 mm, tube rotation time 0.5 ms. Second, the PET scan was performed that covered the identical transverse field of view. Acquisition time was 2–3 min per table position. Last, the PET image data sets were reconstructed iteratively by applying the CT data for attenuation correction.

Images were processed and reviewed by a multimodal computer platform (syngo; Siemens), and independently evaluated by two experienced nuclear medicine physicians. The physicians reached a consensus in cases of discrepancy. The quantification of glucose metabolic activity was obtained using the standard uptake value (SUV) normalized to body weight. MTV was defined as the sum of the metabolic volume above the threshold of cut-off values, and TLG was defined as the product of the SUVmean and the MTV. Heterogeneity index (HI) was defined as SUVmax divided by SUVmean.

A connecting outline of the volume of interest was set using a cut-off value of 2.5, 40%SUVmax, 50%SUVmax, 60%SUVmax and 70%SUVmax, and the contour around the target lesion inside the boundaries was automatically produced. SUVmax, SUVmean and MTV of all lesions in each patient are measured, but considering partial volume effect and repeatability, lesions with diameter of less than 10 mm were excluded [21], and bone lesions were only included in CT confirmation. The maximum SUVmax(maximum group), the average SUVmax(mean group), and the median SUVmax(median group) of all lesions in each patient were selected to calculate the corresponding HI by SUVmax/SUVmean, separately. Taking the threshold of 40%SUVmax and 50%SUVmax as examples, HI_40%_ and HI_50%_, HI_40%-A_ and HI_50%-A_, HI_40%-M_ and HI_50%-M_ represent HI at maximum SUVmax, average SUVmax, and median SUVmax of patient with MHBC, respectively.

### Statistical analysis

All analyses were performed using IBM SPSS software version 20.0 (IBM Corp., Armonk, NY, USA) and GraphPad prism, version 5.0(GraphPad Software, San Diego). The primary endpoint was assessed by progression‐free survival (PFS), which was defined by RECIST 1.1. Correlation between the treatment outcome of dual target therapy and PET parameters was estimated by Spearman Rank Test. The optimal cutoff values for PET/CT parameters were determined by time‐dependent survival receiver operating characteristic (ROC) analysis. The survival analyses were then estimated by Kaplan‐Meier method and compared by log‐rank test. *p* < 0.05 is significant, and all p values were two‐sided.

## Results

### Patient and tumor characteristics

**Fig. 1:**
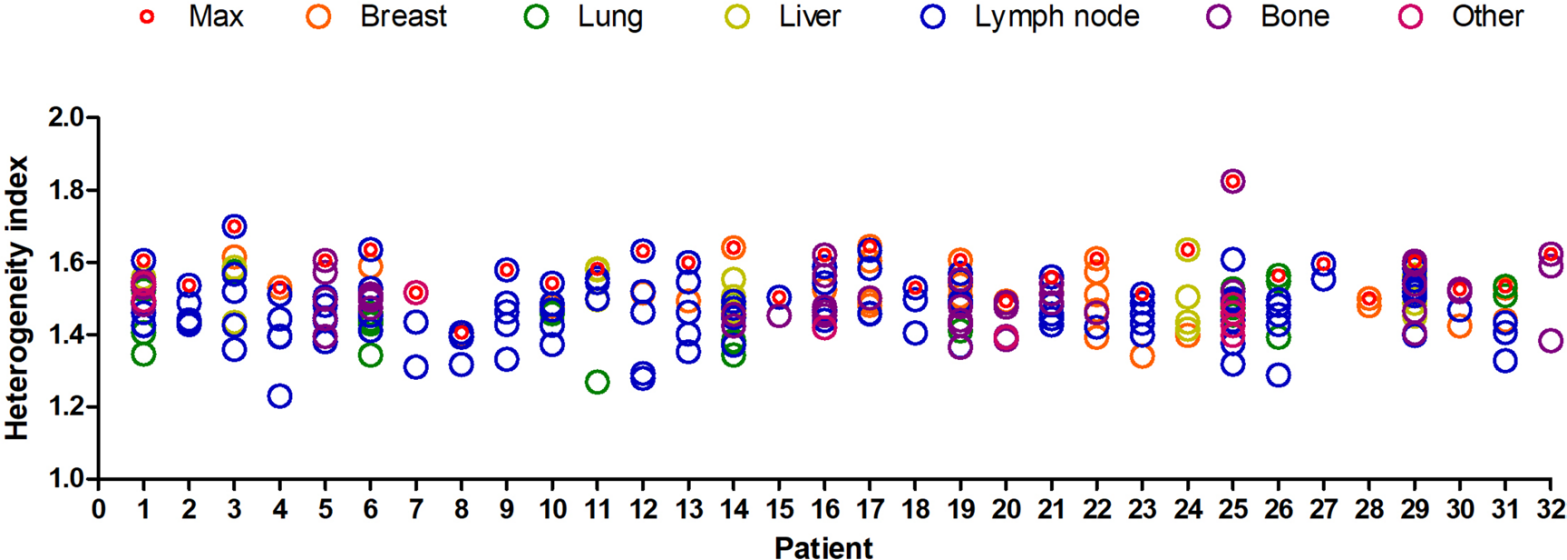
Distribution of metastases per patient and HI_50%_ of all metastases in individual patients

A total of 32 patients with MHBC who received ^18^F-FDG PET/CT scan 1 month prior to dual anti-HER2 therapy with TP, which were included in the analysis. The characteristics of patients are showed in Table 1. The median age is 52 years old (range 32–71). A total of 280 lesions were included in the analysis, including breast, lung, liver, lymph node, bone, and other tissues or organs (including brain, adrenal gland, ovaries, skin/muscles). The HI of each lesion and the largest HI of every patient both are shown in Fig. 1.

**Table 1:** Patients and tumor characteristics

Characteristics	No. of patients	Percent(%)
Age, years		
Median(range)	52(32–71)	
Menopausal status		
Postmenopausal	21	65.63%
Premenopausal	11	34.37%
ER		
+	16	50.00%
-	16	50.00%
PR		
+	9	28.13%
-	23	71.87%
HER2		
+ +	8	25.00%
+ + +	24	75.00%
Primary and Metastatic sites		
Breast	17	53.13%
Lung	12	37.50%
Liver	8	25.00%
Bone	14	43.75%
Viscera	25	78.13%
Lymph node	29	90.63%

Viscera: Liver, lungs, adrenal gland and ovaries

### Correlation between PFS and FDG PET/CT parameters

None of the PET/CT parameters was correlated with disease progression of patient with MHBC treated by the dual target therapy with TP in the mean group and median group. In the maximum group, HI_40%_ was moderately correlated with the progress, and HI_50%_ was strongly correlated with the progress, and other parameters also have no correlation with the progress. The correlation between PET parameters and the progress of patient with MHBC treated by the therapy with TP is shown in Table 2.

**Table 2:** Correlation between pretreatment PET parameters and tumor progression

Threshold	Parameters	Maximum		Mean		Median	
		*r* value	*p* value	r value	*p* value	*r* value	*p* value
2.5	SUVmax	-0.088	0.633	-0.128	0.486	-0.128	0.486
	TLG	-0.205	0.259	-0.073	0.691	-0.073	0.691
	MTV	-0.226	0.213	-0.066	0.721	-0.066	0.721
	HI	-0.209	0.250	-0.117	0.524	-0.117	0.524
	TLG	-0.232	0.201	0.073	0.691	0.073	0.691
40%	MTV	-0.250	0.168	0.066	0.721	0.066	0.721
	HI	-0.426	0.015	0.219	0.228	0.219	0.228
	TLG	-0.225	0.216	0.102	0.578	0.102	0.578
50%	MTV	-0.263	0.146	0.121	0.511	0.121	0.511
	HI	-0.612	< 0.001	0.190	0.298	0.190	0.298
	TLG	-0.195	0.286	0.051	0.781	0.051	0.781
60%	MTV	-0.253	0.162	0.073	0.691	0.073	0.691
	HI	-0.234	0.198	0.212	0.245	0.212	0.245
	TLG	-0.137	0.456	0.044	0.812	0.044	0.812
70%	MTV	-0.171	0.348	0.095	0.605	0.062	0.736
	HI	-0.205	0.261	-0.095	0.605	-0.095	0.605

### Predictive value of treatment outcome

**Fig. 2:**
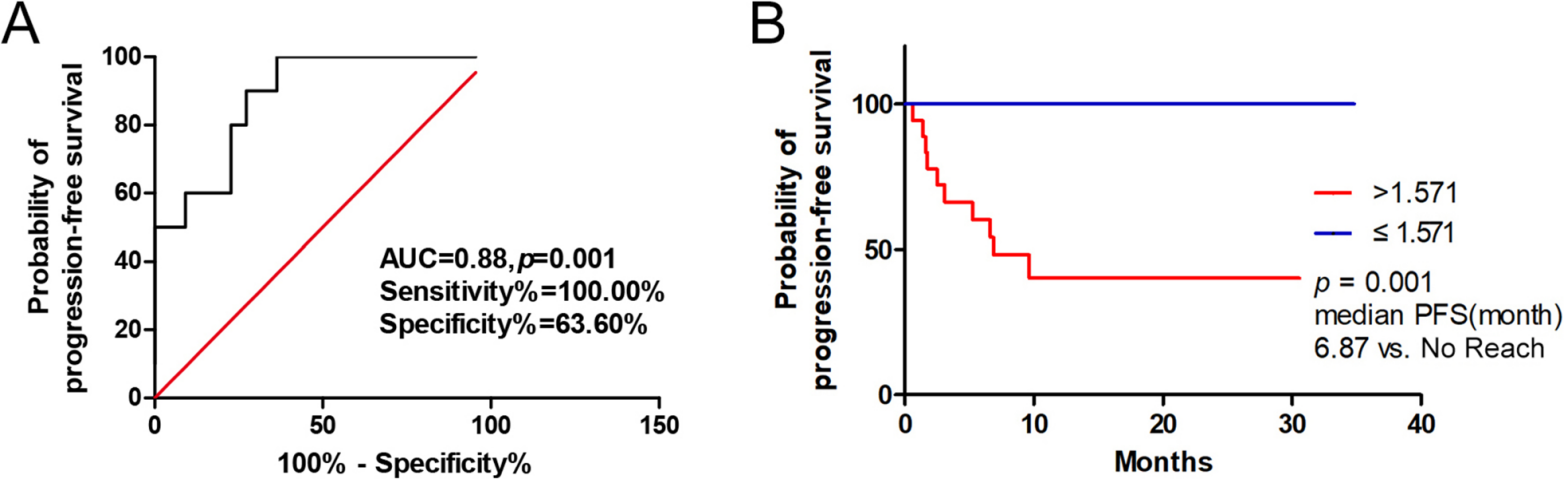
Time-dependent receiver operator characteristics curves for HI_50%_ (**A**); Kaplan–Meier curves of PFS stratified by HI_50%_ (**B**)

In order to further investigate the value of HI_40%_, HI_50%_ in predicting PFS of patients with MHBC treated by the therapy with TP. The optimal cutoff values of HI_40%_ and HI_50%_ for PFS were calculated by time-dependent ROC analysis, and the results were 1.836 and 1.571. The areas under the ROC curve (AUC) were also estimated by time‐dependent ROC curves. HI_50%_ as PET/CT parameter, the AUC of ROC curves was 0.88, which showed a good predictive value, and the sensitivity and specificity were 100.00% and 63.60% as showed in Fig. 2A; HI_40%_ as PET/CT parameter, the AUC of ROC curves was 0.72, which showed a moderate predictive value, and the sensitivity and specificity were 80.00% and 68.20% (see Fig. 1A of additional file 1).

**Fig. 3:**
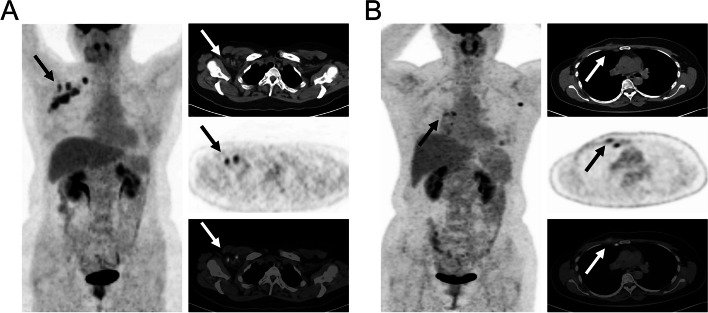
Representative images. **A** A 47‐year‐old female patient with MHBC underwent ^18^F‐FDG PET/CT scan, and the lesion of largest HI is showed by the arrow, the SUVmax = 3.76, SUVmean = 2.48, HI_50%_ = 1.516, and her PFS has not reached within 20.47 months of follow-up; **B** A 51‐year‐old female patient with MHBC underwent ^18^F‐FDG PET/CT scan, the lesion of largest HI_50%_ is showed by the arrow, the SUVmax = 4.83, SUVmean = 3.06, HI = 1.578, and her PFS was 3.09 months

Then, the prognostic value of PET/CT parameters was analyzed by Kaplan‐Meier method. HI_50%_ as PET/CT parameter, the median PFS of patients with high HI_50%_ (> 1.571, *p* = 0.001) was 6.87 months, and the median PFS with low HI50% did not reach in follow-up period, which showed in Fig. 2B. HI_40%_ as PET/CT parameter, the median PFS of patients with high HI_40%_ (> 1.836, *p* = 0.006) was also 6.87 months, and the median PFS with low HI40% also did not reach in follow-up period (see Fig. 1B of additional file 1). The typical PET/CT image of patient with MHBC was shown in Fig. 3.

## Discussion

In this retrospective study based on baseline pretreatment ^18^F-FDG PET/CT imaging to early allow prediction of treatment outcomes with TP for MHBC, we determined that the intra-tumoral HI based on PET/CT parameters was a predictive factor for PFS in patients MHBC treated by TP, and low HI at baseline was significantly associated with improved PFS. As far as we know, this is the first study to evaluate the predictive ability and prognostic significance of ^18^F-FDG PET/CT in patients with MHBC treated by TP, which provides potential clinical value for clinical individualized treatment.

In order to accurately predict the prognostic factors of dual target therapy for MHBC, Kazuhiro Araki et al. showed that baseline absolute lymphocyte counts were a predictive factor for PFS in MHBC treated by TP [22], but infection and prior chemotherapies both may affect baseline levels of absolute lymphocyte counts, therefore, the absolute lymph nodes in predicting PFS of patients with MHBC is controversial. Meanwhile, Koji Takada et al. also showed that tumor-infiltrating CD8 to FOXP3 lymphocyte ratio may predict treatment responses of dual target combination therapy [5], but which is invasive and difficult to be accepted by patients. Connolly et al. showed that the early changes in tumor maximum standardized uptake value corrected for lean body mass (SULmax) of PET/CT parameter predict pathologic complete response to neoadjuvant TP in stage II or III of patients with estrogen receptor-negative and HER2-positive breast cancer [23]. Therefore, we tried to investigate pretreatment ^18^F‐FDG uptake heterogeneity predicts the outcome of patients with MHBC treated by TP.

^18^F-FDG PET/CT imaging, as the most common examination method for tumor patients, is also applied to the diagnosis, staging, monitoring and efficacy prediction of patients with breast cancer [20, 24, 25]. The most common PET/CT parameters, including SUVmax, MTV and TLG were used in various clinical scenarios [26–28]. Unfortunately, there is no correlation between above common parameters and PFS of patients with MBC treated by TP in this study. As mentioned above, HI has been shown to have good prognostic efficacy in various tumors in many studies [16–19]. In the early stage, we tried to predict the treatment results of first-line chemotherapy in patients with metastatic triple negative breast cancer with HI from pretreatment ^18^F-FDG, and obtained satisfactory result [20]. And ^18^F-FDG uptake heterogeneity was applied to reflect spatial tumor heterogeneity among metastases, and baseline HI-intra and HI-inter could both predict the treatment efficacy of pyrotinib in patients with HER2 positive BC [29].

Gradient PET/CT parameters are often used for clinical correlation and predictive analysis [21, 30]. In this study, we analyzed the relationship between gradient PET/CT parameters and the progress of HMBC. And the results show that only the HI_40%_ and HI_50%_ had significant negative correlations with the progression of HMBC after double target therapy. The main reasons may be as follows: as the cut-off value for distinguishing benign and malignant pulmonary nodules in SUVmax, 2.5 was proposed by a small sample size study in 1993 [31], which cannot be widely used for other tumors, including MHBC. 60% and 70% SUVmax fixed threshold segmentation may lose more tumor cell metabolic data on the one hand, and may reduce volume of interesting (VOI) and increase partial volume effect on the other hand.

Some studies show that there are differences in individual treatment responses, which has been attributed to intra-tumoral heterogeneity, including tumor cell metabolism, proliferation, angiogenesis, necrosis, and tumor fibrosis [14]. Other studies show that immune cells in primary tumors is very important for tumor treatment, which can predict the tumor treatment efficacy [32]. Therefore, individual variability in tumor therapeutic response is not only due to the heterogeneity of tumors, but also may be affected by the immune cell infiltration of tumors. In the past, it is generally believed that ^18^F-FDG is taken up by tumor cells in tumor lesions, and the heterogeneity of ^18^F-FDG uptake in tumors was also affected by the number of tumor cells, proliferation, angiogenesis, necrosis and hypoxia. However, the latest research shows that^18^F-FDG is not only taken up by tumor cells, but also by immune infiltrating cells in tumor lesions [33]. Therefore, ^18^F-FDG uptake heterogeneity can more comprehensively show the heterogeneity of tumor cells and immune cells in tumor lesions. This may be one of the reasons why HI can predict PFS early of patient with MHBC in this study, but they still need to be confirmed by corresponding mechanism experiments.

With the rise of artificial intelligence, more and more machine learning algorithms, including Artificial neural network (ANN) [34], Decision tree (DT) [35], K-nearest neighbour (KNN) [36], Logistic regression (LR) [37], Naïve Bayes (NB) [36], Random forest (RF) [38] and Support vector machine (SVM) [39], are used to predict clinical treatment [40, 41]. Although the above machine learning algorithms have their own advantages in the prediction process, they are all too complex and require the assistance of specialized computer engineers, which is not practical in clinical practice. However, the HI mentioned in this study represents the metabolic distribution within the tumor, which is very easy to obtain by dividing SUVmax by SUVmean. At the same time, it is easy for clinicians to understand and accept. Therefore, the clinical practicability of the HI is significantly higher than the above computer learning algorithm. Taking this study as an example, the higher the PET/CT parameter-HI, the worse the dual-target therapy effect be in patients with MHBC, which implies whether clinicians need to increase other supplementary treatments or to improve the effectiveness of therapy or directly switch to second-line treatment.

There are still some shortcomings in this study. First, some HER2 imaging agents have been studied in clinic, including ^89^Zr-trastuzumab [42] and ^68^ Ga-HER2 affinity [43],^18^F-nanobody [44] etc. However, which are not mature and still in the stage to evaluate whether tumor uptake of HER2 imaging can distinguish HER2-positive from HER2-negative breast cancer. Second, this study is a small sample regression exploration and the number of events is not large. Finally, as the data of HI proposed in this study are mainly distributed between 1 and 2, so that cox proportional hazards analysis was not conducted in this study.

## Conclusion

Our preliminary study showed that HI based on pretreatment ^18^F-FDG uptake may predict the PFS of patients with HER2 positive MBC treated by first line dual target therapy. It could be useful to identify patients with HER2 positive MBC who would benefit from dual target therapy and provide theoretical support for individualized treatment. Although this preliminary study outlines the potential prognostic performance of HI, findings need to be further evaluated and confirmed by large, prospective clinical research.

### Supplementary Information

**Additional file 1: Figure 1.** Time-dependent receiver operator characteristics curves for HI_40%_ (A); Kaplan–Meier curves of PFS stratified by HI_40%_ (B).

## Data Availability

The datasets used and analyzed during the current study are available from the corresponding author on reasonable request.

## Abbreviations

^18^F‐FDG: ^18^F‐fluorodeoxyglucose
HER2: Human epidermal growth factor receptor 2
MHBC: Metastatic HER2 positive breast cancer
TP: Trastuzumab and Pertuzumab
PET/CT: Positron emission tomography/computed tomography
ROI: Region of interesting
SUV: Standard uptake value
TLG: Total lesion glycolysis
MTV: Metabolic tumor volume
HI: Heterogeneity index
ROC: Receiver operating characteristic
AUC: Area under curve
PFS: Progression free survival
RECIST1.1: Response Evaluation Criteria in Solid Tumors 1.1
