# Supplementary figures and images for: Pretreatment 18F‐FDG uptake heterogeneity may predict treatment outcome of combined Trastuzumab and Pertuzumab therapy in patients with metastatic HER2 positive breast cancer
Source: Cancer Imaging. 2023 Sep 19;23:90. doi: 10.1186/s40644-023-00608-0 (PMC10510219; doi:10.1186/s40644-023-00608-0)

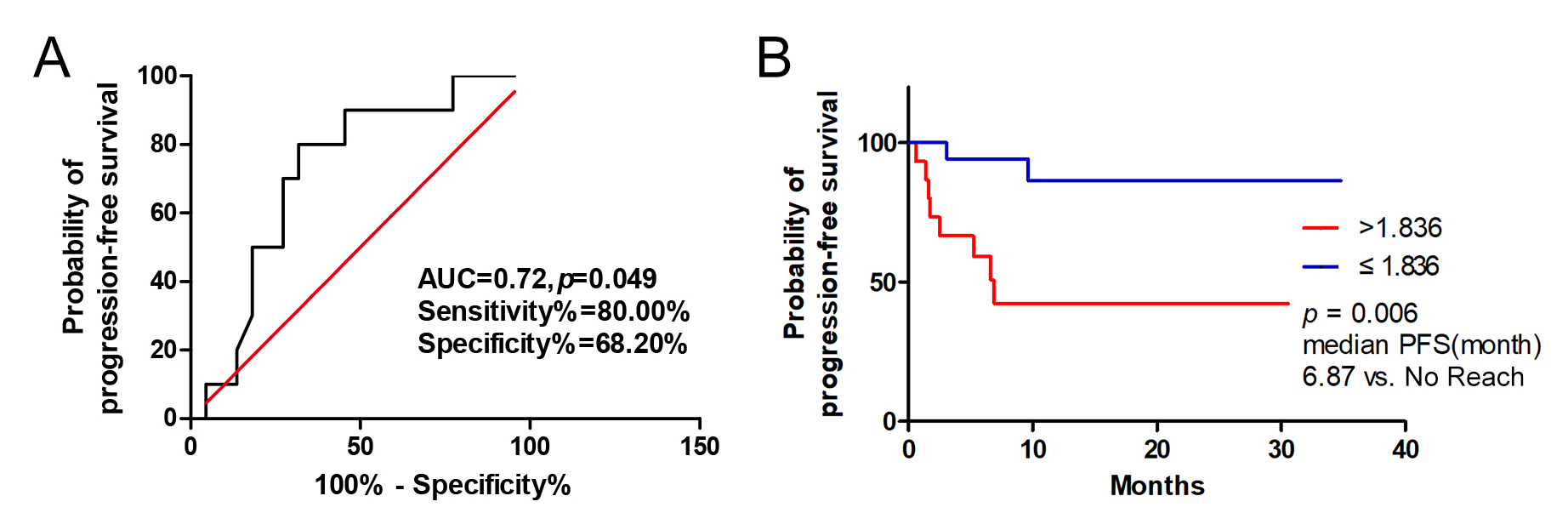

Supplement: Supplementary file 1 — Additional file 1: Figure 1. Time-dependent receiver operator characteristics curves for HI40% (A); Kaplan–Meier curves of PFS stratified by HI40% (B). [file 40644_2023_608_MOESM1_ESM.jpg]
